# Sphingolipids containing very long-chain fatty acids regulate Ypt7 function during the tethering stage of vacuole fusion

**DOI:** 10.1016/j.jbc.2024.107808

**Published:** 2024-09-21

**Authors:** Chi Zhang, Jorge D. Calderin, Logan R. Hurst, Zeynep D. Gokbayrak, Michael R. Hrabak, Adam Balutowski, David A. Rivera-Kohr, Thomas D.D. Kazmirchuk, Christopher L. Brett, Rutilio A. Fratti

**Affiliations:** 1 Department of Biochemistry, University of Illinois Urbana-Champaign, Urbana, Illinois, USA; 2 Department of Biology, Concordia University, Montreal, Quebec, Canada; 3 Center for Biophysics & Quantitative Biology, University of Illinois Urbana-Champaign, Urbana, Illinois, USA

**Keywords:** Elo3, HOPS, SNARE, Ypt7, Vps33

## Abstract

Sphingolipids are essential in membrane trafficking and cellular homeostasis. Here, we show that sphingolipids containing very long-chain fatty acids (VLCFAs) promote homotypic vacuolar fusion in *Saccharomyces cerevisiae*. The elongase Elo3 adds the last two carbons to VLCFAs that are incorporated into sphingolipids. Cells lacking Elo3 have fragmented vacuoles, which is also seen when WT cells are treated with the sphingolipid synthesis inhibitor Aureobasidin-A. Isolated *elo3*Δ vacuoles show acidification defects and increased membrane fluidity, and this correlates with deficient fusion. Fusion arrest occurs at the tethering stage as *elo3*Δ vacuoles fail to cluster efficiently *in vitro*. Unlike HOPS and fusogenic lipids, GFP-Ypt7 does not enrich at *elo3*Δ vertex microdomains, a hallmark of vacuole docking prior to fusion. Pulldown assays using bacterially expressed GST-Ypt7 showed that HOPS from *elo3*Δ vacuole extracts failed to bind GST-Ypt7 while HOPS from WT extracts interacted strongly with GST-Ypt7. Treatment of WT vacuoles with the fluidizing anesthetic dibucaine recapitulates the *elo3*Δ phenotype and shows increased membrane fluidity, mislocalized GFP-Ypt7, inhibited fusion, and attenuated acidification. Together these data suggest that sphingolipids contribute to Rab-mediated tethering and docking required for vacuole fusion.

The lipid composition of cell membranes is extremely complex, and specific configurations inherent to each compartment bilayer provide physical and chemical properties required for organelle function (1). Controlling the stoichiometry of lipids is a key determinant of membrane trafficking events such as endocytosis and organelle fusion, and changes to the ratio of lipids can drastically affects these processes (2, 3). Using *Saccharomyces cerevisiae* as a model system facilitates the study of how lipids are individually modulated, and how the effects of these adjustments can be monitored at the level of specific organelles, such as the lysosome/vacuole. Lysosomes are often viewed as catabolic endpoints for endocytic and autophagic events, but they also serve as a signalling hub to control cellular metabolism or ageing for example, and pathogen clearance through the phagosome-lysosome fusion pathway (4, 5).

Fusion between organelles occurs through a series of stages that are conserved in the trafficking of secretory, endosomal and autophagic membranes (6, 7). In the case of *S. cerevisiae*, the spatiotemporal localization and activity of many proteins and lipids that participate in homotypic vacuole fusion are well understood. In terms of proteins, pre-priming is dependent on the transfer of the ATPase Sec18/NSF from the membrane to inactive *cis*-SNARE complexes *via* its adaptor protein Sec17/α-SNAP (6, 8, 9). Priming occurs when Sec18 hydrolyzes ATP and transfers mechanical force through Sec17 onto the *cis*-SNARE bundle to separate it into its individual components (10). This is followed by tethering through interactions between the late endosome/lysosomal Rab GTPase Ypt7/Rab7 and the HOPS effector complex (11, 12). During docking vacuoles are drawn together to form flattened boundary domain discs where they are in tight contact. At the edge of the boundary disc where vacuoles touch, a “vertex ring” forms composed of membrane microdomains enriched in the proteins and lipids that drive membrane fusion (13, 14, 15, 16, 17). During this stage SNAREs from opposite membranes bundle together in a tight, coiled-coil motif in *trans* that triggers the release of Ca^2+^ from the vacuole lumen (18, 19). Hemifusion occurs when the outer cytoplasmic facing leaflets fuse while leaving the inner leaflets intact (20, 21). This ultimately leads to pore formation between the two organelles and full content mixing to complete a fusion cycle.

In addition to proteins, membrane fusion requires a set of regulatory lipids that are frequently modified by kinases, phosphatase and lipases at specific times throughout the vacuole fusion process (3). Included in this group are the glycerolipids DAG, PA, and the phosphoinositides PI3P, PI4P and PI(4,5)P_2_, and the yeast sterol ergosterol. These lipids co-enrich with fusion machinery proteins in an interdependent manner to form microdomains within the vertex ring during the docking stage. The modification of lipids adds a layer of complexity to the fusion system through changing their stoichiometry in a spatial and temporal manner. As such, the correct ratio of lipids must be organized to progress from one stage to the next. A major way in which these lipids function is through the recruitment of proteins to the vacuole membrane. For instance, PA functions as a negative regulator early in the pathway by sequestering Sec18 from inactive *cis*-SNARE complexes in what we now refer to as the pre-priming stage (6, 9). Sec18 is transferred to *cis*-SNARE bundles after PA is converted to DAG by the phosphatase Pah1, thus allowing SNARE activation/priming to occur. PA serves a second role later in the pathway as a positive regulator through an undefined function (22). One possible function is binding to a second site on the Vam7 PX domain (23). PI3P has multiple roles in vacuole fusion. This lipid recruits the soluble SNARE Vam7, the nucleotide exchange factor Mon1-Ccz1, and HOPS, and regulates the assembly of the vertex ring (23, 24, 25, 26, 27). PI4P and PI(4,5)P_2_ bind HOPS and the latter regulates SNARE priming, actin remodeling, vertex assembly and serves as a substrate for phospholipase C activity (13, 15, 27, 28, 29). PI(3,5)P_2_ was originally identified as positive regulator of vacuole fragmentation and Ca^2+^ release during osmotic shock (30). We later found that it is also needed at low levels for fusion whereas high concentrations inhibits fusion in a manner that is linked to Ca^2+^ transport and vacuole acidification (31, 32, 33).

Most of the effects of regulatory lipids are linked to protein binding. This does not consider the physical effects that lipids have on membranes such curvature and fluidity. There is a wealth of research showing how ergosterol, DAG and PA affect fluidity and curvature (34, 35, 36, 37, 38, 39). In contrast to glycerolipids, there are relatively few studies that investigate the role of sphingolipids in membrane fusion (40, 41, 42). Sphingolipids are important bioactive lipids in mammalian cells, with roughly 40 enzymes contributing to their highly regulated metabolism, and defects in their metabolism are linked to human lysosomal storage diseases. However, how sphingolipids contribute to lysosome morphology and function is not understood (43, 44).

In yeast, very-long-chain fatty acids (VLCFAs) are almost exclusively bound to long chain bases (LCBs) as ceramides or glycosphingolipids. Free VLCFAs in cell extracts are scarcely detectable (45). Baker’s yeast contains three fatty acid elongases (Elo1, Elo2, and Elo3) that catalyze the condensation of a malonyl-CoA unit with a long-chain fatty acyl-CoA at the endoplasmic reticulum. Each of these enzymes has an inherent substrate specificity and unique VLCFA major product, such as Elo3 displaying specificity for producing VLCFAs up to 26-carbons in length (46, 47). In mammalian cells VLCFA production has been linked to processes including phagocytosis, apoptosis, and myelin function, and has been connected to diseases such as ichthyosis, macular degeneration, and myopathy (48, 49, 50). C26 VLCFAs are likely critical for the function of yeast sphingolipids in membranes based on research demonstrating that this particular species of fatty acid is found in the majority sphingolipid species (51). Studies with yeast lacking functional elongase enzymes have uncovered links between sphingolipids and Vps21-related endosomal maturation, vacuolar acidification, and vacuolar morphology/function, but their role as regulatory lipids in membrane fusion remains unclear (52, 53, 54, 55, 56).

When yeast age or are subjected to metabolic stress they form lipid raft-like domains on their vacuoles. These are transient, sub-organellar domains, which phase separate based on differences in lipid and protein composition that alter membrane properties such as fluidity and thickness. These domains are highly enriched in sterols, sphingolipids and other lipids with saturated acyl chains, and display liquid-ordered (L_o_)-like properties (57, 58, 59). Thus, it is likely that sphingolipids colocalize with ergosterol in the vertex ring to regulate fusion. It is also likely that vacuolar sphingolipids affect other vacuolar functions including acidification by the V-ATPase (60).

In this study we present evidence that sphingolipids generated *via* Elo3 contribute to the membrane fluidity, morphology and homotypic fusion of vacuoles. Deleting *ELO3* disrupts tethering and docking stages of vacuole fusion by hindering Ypt7 enrichment at vertex sites. Thus, we speculate that C26 VLCFA containing sphingolipids (C26-SL) contribute to assembly of specialized membrane microdomains necessary for regulating the protein machinery that drives vacuole fusion.

## Results

### Yeast cells lacking Elo3 contain fragmented vacuoles

**Figure 1:**
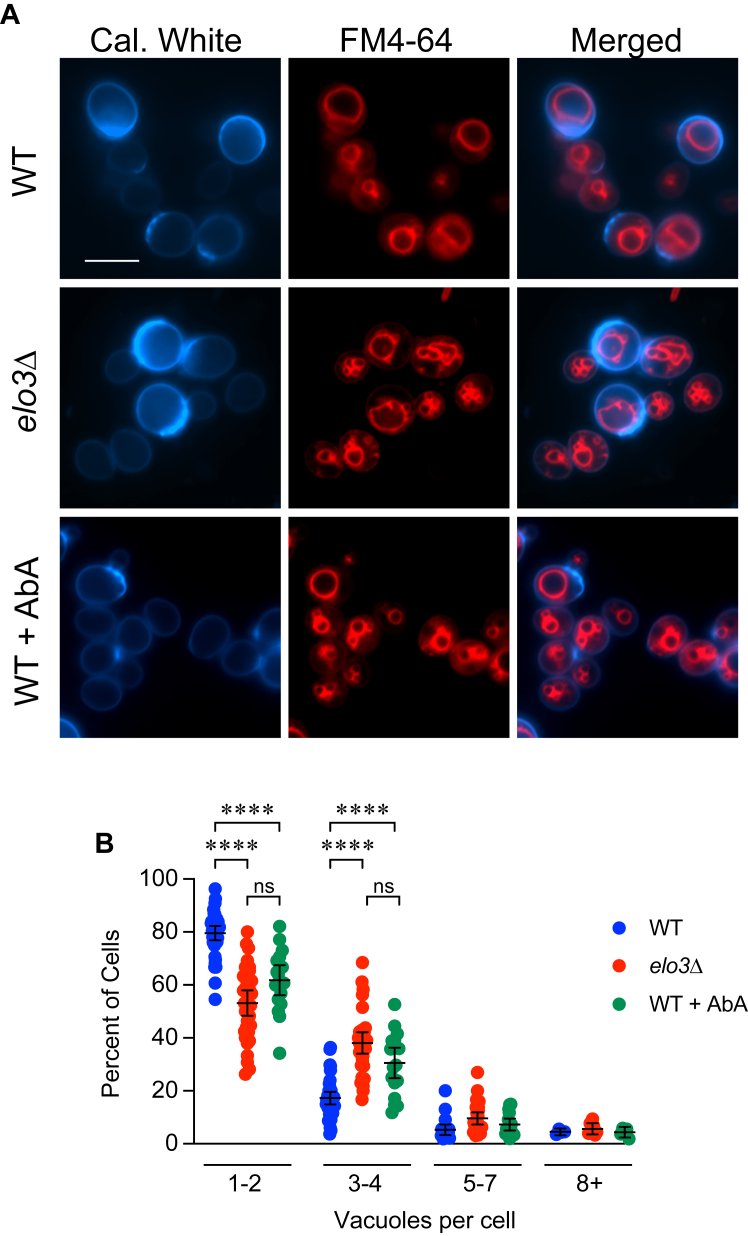
**Vacuole morphology is perturbed when sphingolipid synthesis is interrupted.***A*, WT and *elo3*Δ cells were grown overnight, back-diluted to OD_600_ ∼0.7 in fresh YPD and grown for 2 h. WT cells were then treated with 0.125 μg/ml aureobasidin A (AbA) and incubated for 4 h. Cells were incubated with 5 μM FM4-64 to label vacuoles. Cell walls were stained calcofluor white. Images are representative of three separate trials. Scale bar: 5 μm. *B*, Vacuole fragmentation was quantified using data from *A*. Data was graphed as column scatter plots with error bars representing mean ± 95% CI. Significance was determined with one-way ANOVA for multiple comparisons [F (11, 249) = 186.5, ∗∗*p* < 0.0001]. (n = 3). Tukey’s multiple comparison test was performed for each cluster bin for individual *p* values. Ten fields containing ≥20 cells were counted per condition per experiment. Each data point represents a single field. ∗∗∗∗*p* < 0.0001; ns, not significant.

To examine the role that C26-SL play in vacuole fusion we deleted *ELO3* from *S. cerevisiae,* and vacuole morphology was examined in live cells. WT yeast grown to logarithmic phase in YPD were round, and most cells had one or two vacuoles (Fig. 1, *A* and *B*). However, *elo3*Δ cells had fragmented and misshapen vacuoles. We next visualized WT yeast after treatment with a known inhibitor of sphingolipid biosynthesis. Aureobasidin A (AbA) was used to block inositol-phosphoceramide (IPC) synthesis (61, 62). We found that AbA mimicked the vacuole fragmentation pattern seen in *elo3*Δ cells (Fig. 1, *A* and *B*). In addition, inhibiting of LCB synthesis with myriocin had effects similar to AbA (not shown) (63, 64). These results were important when we considered that IPC is enriched in yeast vacuoles (65), and that IPC along with ceramide are thought to be required for proper vacuole morphology and function (66).

### *elo3*Δ vacuoles have attenuated fusion activity

**Figure 2:**
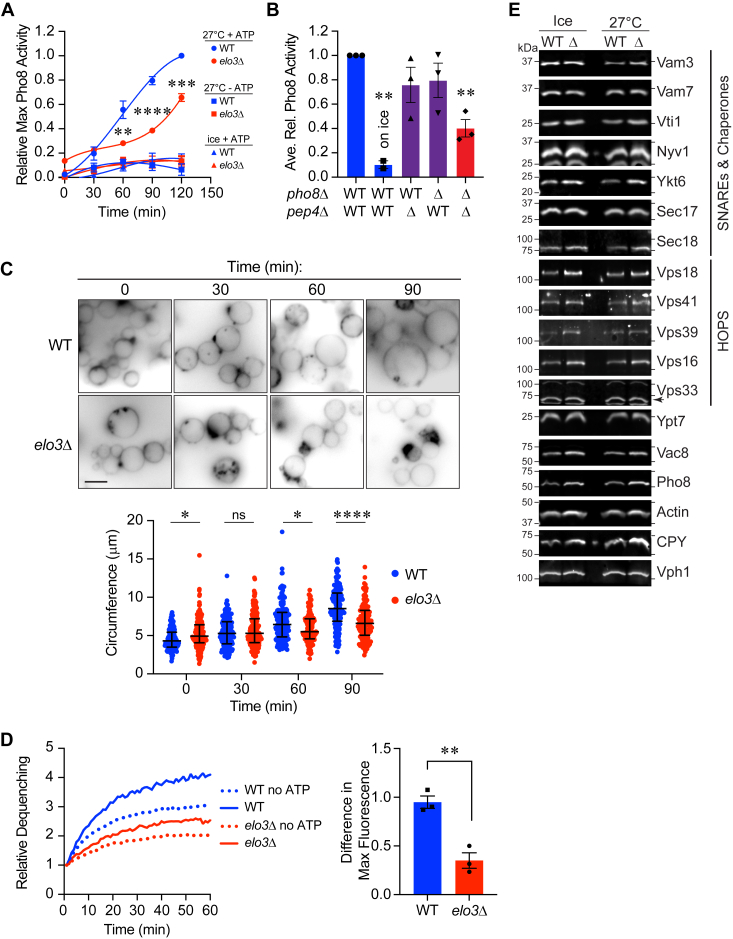
**Vacuoles from *elo3*Δ cells are fusion impaired.***A*, time course of fusion for WT or *elo3*Δ vacuoles. Fusion activity was expressed as the percentage of WT activity. Significance was determined using one-way ANOVA for multiple comparisons for each time point between strains [F (5, 12) = 15.62; ∗∗∗∗*p* < 0.0001]. Tukey’s multiple comparison test was used for individual *p* values (n = 3). Error bars represent mean ± SE. ∗∗*p* < 0.01. ∗∗∗*p* < 0.001, ∗∗∗∗*p* < 0.0001. *B*, endpoint fusion (90 min) reactions were performed with combinations of WT and *elo3*Δ (Δ) vacuoles. Significance was determined using one-way ANOVA for multiple comparisons with WT/WT as a control [F (4, 9) = 10.07; ∗∗*p* = 0.0022]. Dunnett’s test for multiple comparison was used for individual *p* values. Error bars represent mean ± SE. (n = 3). ∗∗*p* < 0.01. *C*, measurement of vacuole diameters after fusion. WT and *elo3*Δ vacuoles were incubated for the indicated times at 27 °C and images were taken by fluorescence microscopy. Diameters of individual vacuoles in clusters were measured using ImageJ. Data was graphed in a scatter plot of the pooled data. Significance was determined using one-way ANOVA for multiple comparisons [F (7, 1288) = 53.22 *p* < 0.0001]. Tukey’s test for multiple comparisons was used for individual *p* values. The bars represent the median with upper and lower quartiles. (n = 3). ∗*p* < 0.05, ∗∗∗∗*p* < 0.0001. Scale bar: 5 μm. *D*, lipid mixing experiments were performed with WT or *elo3*Δ vacuoles. Fluorescence (ex = 544 nm, em = 590 nm) was measured every 60 s after the addition of ATP. *Left*, A representative run of the experiment. *Right*, Quantitation of three experiments calculated by measuring the difference in fluorescence between no ATP and ATP for WT and *elo3*Δ at the end of the assay. Error bars represent mean ± SE (n = 3). Data was analyzed using an unpaired two-tailed *t* test ∗∗*p* < 0.01. *E*, Western blots of WT and *elo3*Δ vacuoles that were incubated on ice or at 27 °C for 1 h. Vacuole extracts were resolved by SDS-PAGE and transferred to nitrocellulose for immunoblotting using antibodies against the specified proteins.

To examine how the lack of Elo3 affected homotypic fusion, vacuoles were isolated from WT and *elo3*Δ strains. Vacuoles were incubated under fusion conditions and *in vitro* fusion was monitored *via* Pho8 phosphatase activity upon content mixing. This showed that *elo3*Δ vacuole fusion only reached ∼50% relative to WT vacuoles by 90 min (Fig. 2*A*). The fusion of *elo3*Δ seemed to accelerate by 120 min, however, overall fusion was still suppressed.

We next asked if changes in membrane composition were needed on both sets of vacuoles to inhibit fusion. To do this we mixed fusion partners from WT and *elo3*Δ backgrounds. Fusion that only contained *elo3*Δ vacuoles was inhibited (Fig. 2*B*, red). However, when fusion partners were mixed from WT and *elo3*Δ backgrounds the fusion defect was mostly rescued (Fig. 2*B*, purple), indicating that the Elo3-dependent factor(s) important for fusion were sufficient on one side of the fusion pair. Together this data suggested that C26-SL were positive regulators of homotypic vacuolar fusion. To further verify that *ELO3* impacted vacuole fusion, we took visual measurements of vacuole circumferences at different time points during fusion (67). At each time point vacuoles were placed on ice and stained with FM4-64 prior to imaging. At the start of fusion (T = 0 min) we observed that the average circumference of *elo3*Δ vacuoles was slightly bigger than the WT. As fusion progressed, we measured a significant increase of WT vacuole circumference *versus elo3*Δ demonstrating vacuole fusion was indeed attenuated in the mutant (Fig. 2*C*).

In previous studies we found that mutating the zero-layer Gln of the Q-SNARE Vam7 to an Arg inhibited vacuole content mixing but not hemifusion (68, 69). The same arrest point was found with the Vam3-CCIIM mutant in which its transmembrane domain was replaced with the isoprenylation sequence from Ykt6 (70). In both cases content mixing was restored when membrane fluidity was increased with chlorpromazine. In contrast, increasing fluidity reduced the efficacy of WT Vam7 in supporting fusion. Together this suggested that lipid composition sets an optimal fluidity and curvature to match the force exerted by SNARE complex formation.

Because sphingolipids are known to affect membrane fluidity through the stabilization of membrane microdomains containing cholesterol we next tested if *elo3*Δ vacuoles were arrested before or after hemifusion. To measure hemifusion we isolated vacuoles from WT and *elo3*Δ cells and labeled them with rhodamine B-conjugated phosphatidylethanolamine (Rh-PE) at a self-quenching concentration (20, 21). Labeled vacuoles were then incubated with an excess of unlabeled vacuoles. As the outer leaflets fuse, Rh-PE is diluted leading to its dequenching. This is kinetically separated from full content mixing to show that the signal was due to hemifusion (71). Using this method, we found that *elo3*Δ vacuoles were impaired in hemifusion (Fig. 2*D*). The VLCFAs present in sphingolipids have been suggested to help stabilize highly curved membranes, which promotes membrane fusion (72, 73), which may help explain their contribution to fusion events. Furthermore, stabilization of membrane curvature is needed for hemifusion. From these data we deduced that 26C-SLs regulated a stage before hemi-fusion.

Protein sorting to the vacuole through the AP3 or CPY pathways culminates in membrane fusion, consequently, defects in vacuole homotypic fusion can be due to defective protein sorting. Thus, we checked if the reduced fusion of *elo3*Δ vacuoles was due to altered protein sorting. We used isolated WT and *elo3*Δ vacuoles and incubated for 1h at 27 °C or on ice under fusion conditions. This was to see if there were any changes in degradation or protein modification. As seen in Fig. 2*E* we did not observe any significant difference between strains under either condition. This told us that differences in fusion were not due to the absence of a fusion-driving protein. This is consistent with work by others showing that CPY sorting to the vacuole was not affected by deleting *ELO3* (74).

### Membrane fluidity is altered in the absence of C26-SL

Lipid rafts at the plasma membrane are characterized by their increased rigidity and thickness in relation to the surrounding membrane environment (75). These characteristics are due in part to tight packing of sphingolipids with cholesterol. Although not identical to classically defined rafts, yeast vacuoles form ergosterol-rich microdomains at the vertex contact points of docked vacuoles (13, 16, 17). Here we tested whether the absence of C26 VLCFAs affected membrane fluidity. To measure membrane fluidity, we used merocyanine 540 (MC540) (76, 77, 78). MC540 has a peak emission at 624 nm when inserted in gel-phase membranes and its fluorescence shifts to 585 nm when it intercalates in liquid crystalline membranes with increased fluidity. Thus, increased fluidity would correspond with increased fluorescence at 584 nm. We chose MC540 because its fluorescence was not affected by small molecule inhibitors that we commonly use, including dibucaine, an anesthetic that increases membrane fluidity (79). The fluorescence of other probes used to measure fluidity, *e.g.*, TMA-DPH overlaps with dibucaine making it difficult to use in these experiments.

**Figure 3:**
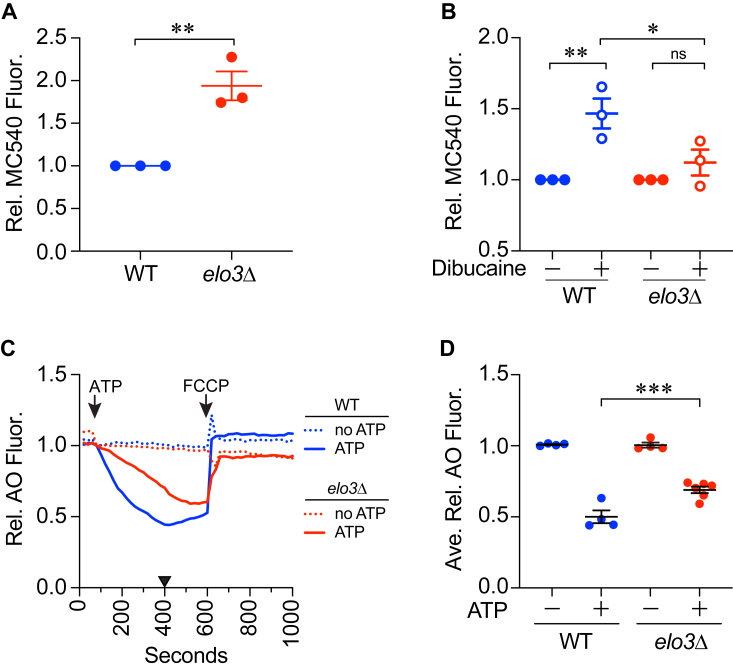
**C26-SL are important for membrane fluidity and acidification.***A*, vacuoles were mixed with MC540 in PS buffer. MC540 fluorescence was measured (ex = 530 nm, em = 585 nm) and intensities were normalized to the WT set to 1. Data was analyzed using an unpaired two-tailed *t* test. Error bars represent mean ± SE (n = 3). ∗∗*p* < 0.01. *B*, WT vacuole fluidity was determined as described above in the presence or absence of 500 μM dibucaine in 0.5% ethanol. Here, changes in fluorescence were normalized to each strain that was set to 1. Changes in fluorescence was analyzed using one-way ANOVA for multiple comparisons with no treatment (0 μM dibucaine/0.5% ethanol) as a control [F (4, 14) = 14.87; ∗∗∗∗*p* < 0.0001]. Dunnett’s multiple comparison test was used for individual *p* values. Error bars are mean ± SE. (n = 3). ∗∗*p* < 0.01. *C*, vacuole acidification was determined by measuring changes in AO fluorescence at 520 nm. Fluorescence decreased as vacuoles were acidified. Reactions were incubated with or without ATP and fluorescence was measured every 20 s. The protonophore FCCP was added at 500 s to collapse the proton gradient. Fluorescence was normalized to initial intensities at the time of adding ATP and set to 1. *D*, quantitation of multiple experiments shown in *C*. Fluorescence values at 400 s (WT peak acidification) were averaged for each strain. Significance was measured using one-way ANOVA for multiple comparisons [F (3, 14) = 80.30, ∗∗∗∗*p* < 0.0001]. Tukey’s *post hoc* multiple comparisons test was used for individual *p* values. Error bars represent mean ± SE. (n > 4). ∗∗∗*p* < 0.001.

In Fig. 3 we show MC540 fluorescence at 585 nm with WT and *elo3*Δ vacuoles. We found that MC540 fluorescence of *elo3*Δ vacuoles was significantly higher relative to the WT indicating that the lack of C26 VLCFAs increased fluidity (Fig. 3*A*). This was in accord with the role of SL at the plasma membrane and the formation of lipid rafts. To verify that we were measuring differences in fluidity, we tested vacuoles in the presence of the known fluidizer dibucaine and found that it increased the MC540 fluorescence of WT vacuoles suggesting that the anesthetic significantly increased membrane fluidity (Fig. 3*B*). However, dibucaine treatment of *elo3*Δ vacuoles did not further increase fluorescence. It is likely that under the current setup MC540 could not detect further increases in fluidity with *elo3*Δ vacuoles. Nevertheless, this showed that the lack of C26-VLCFAs increased the fluidity of *elo3*Δ vacuoles.

### Vacuole acidification is reduced in *elo3*Δ vacuoles

We next asked if deleting *ELO3* affected another SL-dependent vacuolar function such as V-ATPase activity. The V-ATPase is composed of the integral membrane complex V_O_ and the soluble V_1_ complex (80). Others have found that regulating sphingolipid homeostasis *via* Orm1 and Orm2 is needed for vacuole acidification as measured by ATPase activity (81). They found that the ATPase activity of was reduced by ∼30% in *orm1*Δ *orm2*Δ cells. Here we tested if isolated vacuoles could become acidified using a real-time acridine orange (AO) fluorescence shift assay (82, 83). AO becomes trapped in acidic compartments due to its protonation and dimerization. Monomeric AO fluoresces at 520 nm whereas protonated AO forms dimers and fluoresces at 680 nm. Thus, a decrease in fluorescence at 520 nm serves as a reporter of vesicle acidification. In Fig. 3*C* we show that upon the addition of ATP, AO fluorescence in the control reaction decreased as protons entered the lumen. Using this assay, we found that vacuoles isolated from the *elo3*Δ strain have significantly impaired proton-pumping ability compared (Fig. 3, *C* and *D*). This is consistent with the previous report showing a positive role for SL content in V-ATPase function and vacuole acidification.

### Ergosterol and PI3P are not affected by the loss of C26-SL

In order to narrow when C26-SL were needed for fusion we started by testing SNARE activation by Sec18 and Sec17 (10). We found that there was little difference between WT and *elo3*Δ vacuole SNARE priming (not shown), suggesting that the role of C26-SL was between tethering/docking and hemifusion. To monitor tethering/docking and vertex ring formation we used fluorescent microscopy as described (13). During tethering/docking, the proteins and lipids that drive fusion accumulate at vertex sites in an interdependent manner. In other words, regulatory lipids are needed for proteins to accumulate at vertices and *vice versa*.

**Figure 4:**
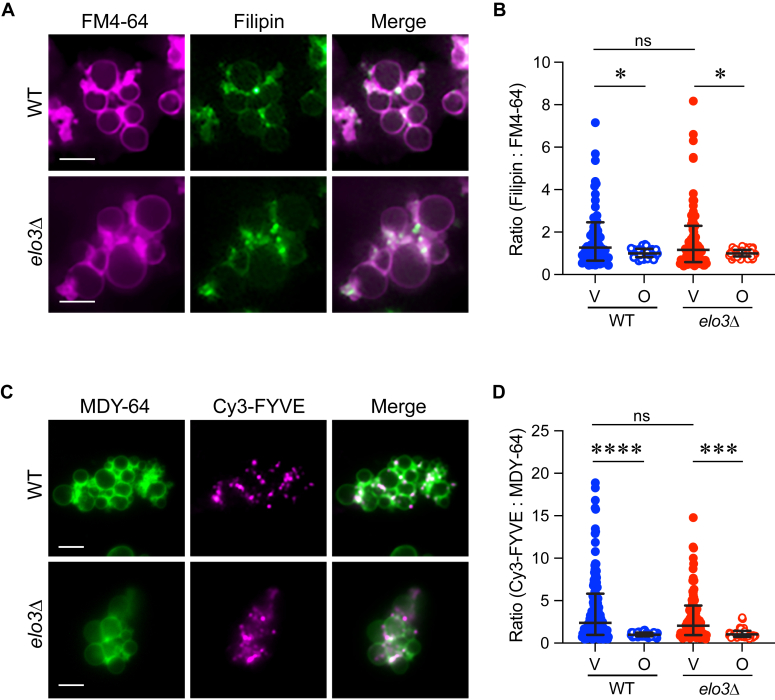
**Vertex enrichment of ergosterol and PI3P does not require C26-SL.***A*, docking reactions using filipin to mark ergosterol. FM4-64 was added at the end of the reaction and docked vacuoles were visualized by fluorescence microscopy. Scale bars: 5 μm. *B*, quantitation of ratiometric fluorescence intensities for vertices (V) and outer edge (O) in panel A. The data points were pooled from multiple experiments where 15 to 20 clusters with ≥10 vacuoles per cluster were analyzed per experiment (n > 400 vertices for each strain; n > 200 outer edge measurements for each strain). Error bars represent geometric means ± geometric SD (n = 3). Significance was measured using one-way ANOVA for multiple comparisons [F (3, 452) = 24.71, ∗∗∗*p* < 0.0001]. Tukey’s *post hoc* multiple comparisons test was used for individual *p* values. ∗*p* < 0.05; ns, not significant. *C*, docking reactions of purified vacuoles were performed in the presence of Cy5-FYVE to mark PI3P. MDY-64 was added at the end of the reaction and docked vacuoles were visualized using fluorescence microscopy. Scale bars: 5 μm. *D*, Quantitation of ratiometric fluorescence intensities for Cy5-FYVE at V and O in panel C as described in A. Error bars represent geometric means ± geometric SD (n = 3). Significance was measured using one-way ANOVA for multiple comparisons [F (3, 291) = 6.152, ∗∗∗*p* = 0.0005]. Tukey’s *post hoc* multiple comparisons test was used for individual *p* values. ∗∗∗∗*p* < 0.0001; ∗∗∗*p* < 0.001 ns, not significant.

The formation of raft-like domains in yeast membranes is strongly influenced by the amount of sphingolipids and ergosterol (57, 59). Furthermore, ergosterol enrichment at vertices is needed for fusion (13, 84). Thus, we tested whether the lack of C26-SL would affect the vertex localization of ergosterol. Using sub-inhibitory amounts of the fluorescent macrolide filipin III, which binds to ergosterol, we compared its localization on WT and *elo3*Δ docked vacuoles (Fig. 4, *A* and *B*). As previously shown, filipin localized to the vertices of docked WT vacuoles indicating that ergosterol was enriched in microdomains. When *elo3*Δ vacuoles were examined, we found no significance difference in vertex enrichment *versus* WT, suggesting that ergosterol accumulation at vertex sites was independent of C26-SL.

Because ergosterol localization was unaffected on *elo3*Δ vacuoles we asked if another regulatory lipid was also unaffected. To do this we used the FYVE domain from human HRS as a probe for PI3P (13, 85). Recombinant GST-FYVE was conjugated to Cy3 and used in vacuole docking reactions at a sub-inhibitory concentration. We found that both WT and *elo3*Δ accumulated comparable amounts of Cy3-FYVE at vertex sites (Fig. 4, *C* and *D*). Altogether, the data suggests that yeast C26-SL do not strongly affect the localization of other regulatory lipids. While ergosterol and PI3P localization were not affected on *elo3*Δ vacuoles, it is still possible that their increased fluidity was sufficient to attenuate fusion efficiency.

### The tethering and docking steps are altered on *elo3*Δ vacuoles

**Figure 5:**
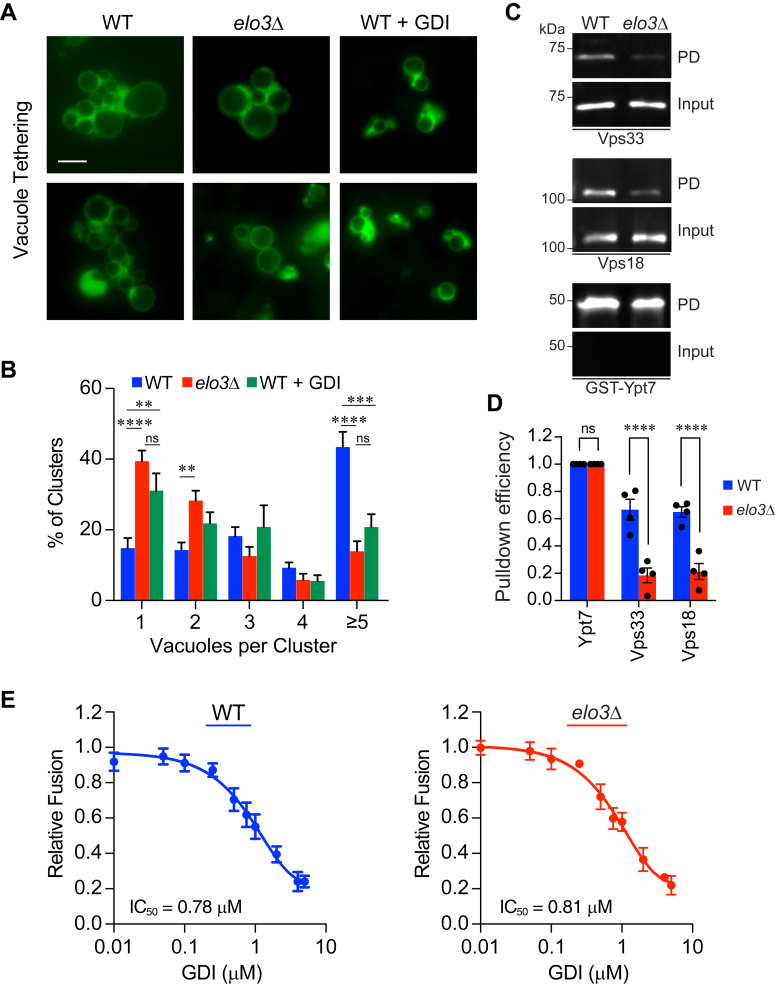
**Vacuole tethering is defective in *elo3*Δ vacuoles.***A*, vacuoles were incubated for 20 min at 27 °C under docking conditions. Following incubation reaction mixtures were placed on ice and stained with MDY64. WT vacuoles were incubated with purified 2 μM GDI to inhibit the Ypt7-dependent tethering of vacuoles. Shown are two examples for each strain and condition. Scale bars: 5 μm. *B*, quantitation of vacuole clustering from *A*. Vacuole clusters (∼300) were counted for each condition. Significance was measured using one-way ANOVA for multiple comparisons [F (14, 295) = 11.75, *p* < 0.0001]. Tukey’s *post hoc* multiple comparisons test was used for individual *p* values. Error bars represent mean ± SE (n = 3). ∗∗*p* < 0.01; ∗∗∗*p* < 0.001; ns, not significant. *C*, co-isolation experiments performed with GST-Ypt7 immobilized on glutathione agarose resin and preloaded with GTP. 6X fusion reactions containing vacuoles from WT or *elo3*Δ were solubilized with TX100 and incubated separately with Ypt7-loaded beads. The amount of Vps33 and Vps18 that was bound to resin and found in the input sample were determined by Western blotting. PD, pulldown. Input lanes show to 10% of the total material added to GST-Ypt7 loaded beads. *D*, quantification and comparison of the GST-Ypt7 pull-down efficiency. Efficiency was calculated by dividing the area of Vps33 or Vps18 pulldown band by the area of the corresponding GST-Ypt7 band in each experiment as determined by ImageJ. Error bars represent mean ± SE (n = 4). Significance was measured using one-way ANOVA for multiple comparisons [F (5, 18) = 56.88 *p* < 0.0001]. Šidák’s test was used for pairwise comparisons between strains and individual *p* values. ∗∗∗∗*p* < 0.0001. ns, not significant. *E*, sensitivity of fusion to GDI. Fusion assays using WT and *elo3*Δ vacuoles were performed with a dose curve of GDI. Each strain was normalized to its own maximum fusion set to 1. Graphpad Prism was used to log transform, normalize and fit the data to a non-linear regression curve to yield IC_50_ values of GDI for vacuole fusion. Error bars represent the mean ± SE. (n = 3).

While examining ergosterol and PI3P accumulation at vertex sites we noted that *elo3*Δ vacuoles formed clusters with fewer vesicles. When quantified, we found that most WT vacuole clusters contained five or more vesicles (Fig. 5, *A* and *B*). In comparison *elo3*Δ clusters contained fewer vacuoles and ∼40% were found alone. To specifically inhibit the tethering step, we incubated vacuoles under the same conditions but in the presence of purified Gdi1, a guanine-nucleotide dissociation inhibitor (GDI) that extracts GDP-bound Rabs from membranes (86, 87). We found that adding GDI to WT vacuoles mimicked the reduced docking efficiency of *elo3*Δ vacuoles. GDI-resistant clusters were those that have moved onto SNARE pairing. Ungermann *et al.*, showed that tethering required Ypt7 and not SNAREs (19). Therefore, we propose that C26-SL regulated the Ypt7-dependent tethering stage.

To confirm that the loss of functional Elo3 significantly impaired the tethering state we performed co-affinity isolation assays of GST-Ypt7 and the HOPS complex (88, 89). To test for Ypt7-HOPS interactions we used recombinant un-prenylated GST-Ypt7 as previously described (90). GST-Ypt7 was bound to glutathione agarose beads and loaded with GTP. Pull-down experiments were performed with 30 μg of vacuolar material from WT or *elo3*Δ membranes. Vacuoles were then solubilized with detergent, mixed with resin-bound GST-Ypt7 and incubated for 2 h at 4 °C. After incubation, the unbound material was removed, and bound Ypt7 complexes were washed prior to elution with reduced glutathione. Samples were resolved by SDS-PAGE and probed for the presence of Vps33, Vps18 and Ypt7 (at 49 kDa for GST-Ypt7) by Western blotting. We found that Vps33 and Vps18 from WT vacuolar extracts bound to GST-Ypt7 (Fig. 5, *C* and *D*). In contrast the extract from *elo3*Δ vacuoles failed to show an interaction between these proteins. Because the Ypt7 used in these experiments was expressed in bacteria and therefore not prenylated, we can conclude that the altered interactions were not due to changes in Ypt7 conformation that could be induced by the *in vivo* conditions found in *elo3*Δ cells. Instead, it appeared that the defect was due to changes in HOPS on *elo3*Δ vacuoles.

Finally, we asked whether the nucleotide binding state of Ypt7 on vacuoles was altered in the absence of C26-SL. As mentioned above, GDI binds to GDP-bound Ypt7 to promote its extraction from membranes. Thus, changes in GDI sensitivity would suggest that Ypt7 on *elo3*Δ vacuoles were shifted to binding more or less GDP relative to WT, which would differentially affect fusion. To test this, we used a concentration curve of GDI in fusion assays containing WT or *elo3*Δ vacuoles. Both curves were normalized to their own maximum fusion in the absence of GDI. We found that the sensitivity of *elo3*Δ vacuole fusion to GDI was identical to that of WT vacuoles (Fig. 5*E*). This suggested that the nucleotide binding state of Ypt7 on *elo3*Δ vacuoles was not affected and that the attenuated fusion in the mutant was due to a factor independent of nucleotide exchange.

### Ypt7 is mislocalized on *elo3*Δ vacuoles

**Figure 6:**
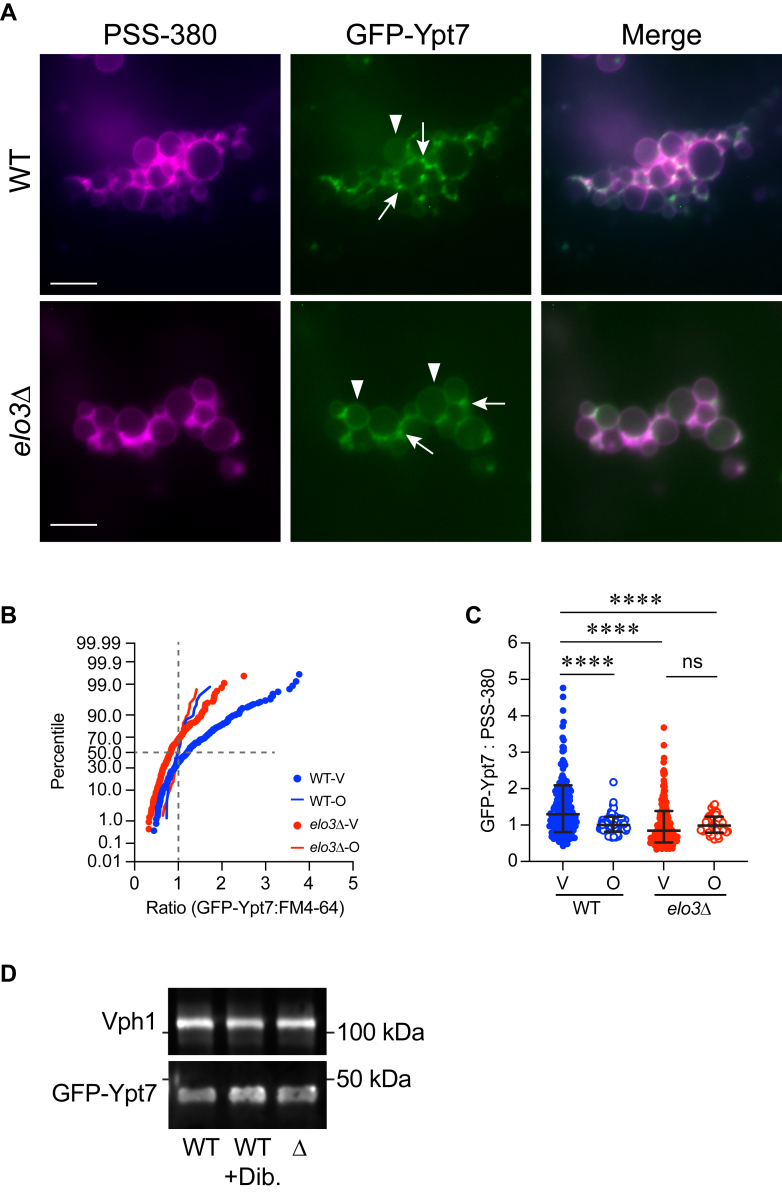
**Ypt7 is mislocalized on *elo3*Δ vacuoles.***A*, WT and *elo3*Δ vacuoles containing GFP-Ypt7 were incubated under docking conditions for 20 min at 30 °C. After incubation, the reactions were placed on ice and labeled with PSS-380. A large cluster of *elo3*Δ vacuoles is shown to see more vertices. Arrows point at representative vertices. Arrowheads point at representative outer edges. Scale bar: 5 μm. *B*, cumulative distribution plot depicting the percentile values of GFP-Ypt7/PSS-380 ratio for vertex (V) and outer edge (O) in WT vs *elo3*Δ vacuoles. Each curve is comprised of pooled data points from three experiments. Each experiment used 10 to 15 clusters with ≥10 vacuoles per cluster (n > 400 vertices for each strain; n > 200 outer edge measurements for each strain) for each condition. (n = 3). *C*, Quantitation of ratiometric GFP fluorescence at vertices (V) and outer edge (O) in *B*. The data points were pooled from multiple experiments and ≥15 to 20 clusters with at ≥10 vacuoles per cluster per experiment for each strain. Error bars represent geometric means ± geometric SD. (n = 3). Significance was measured using one-way ANOVA for multiple comparisons [F (3, 634) = 6.779, ∗∗∗*p* = 0.0002]. Tukey’s *post hoc* multiple comparisons test was used for individual *p* values. ∗∗∗∗*p* < 0.0001; ns, not significant. *D*, Western blot of WT and *elo3*Δ vacuoles. Antibody against Ypt7 was used to show levels of GFP-Ypt7 (∼49 kDa). Antibody against Vph1 was used as a loading control. GFP-Ypt7 levels on WT vacuoles treated with 250 μM dibucaine is also shown.

While Ypt7 is equally abundant on WT and *elo3*Δ vacuoles, the defects in tethering and HOPS interactions suggest that other related events could be dysregulated, including the distribution of Ypt7 itself. To see if Ypt7 properly localized to the vertex ring, we used vacuoles expressing GFP-Ypt7 under control of its native promoter and performed ratiometric fluorescence microscopy with the phosphatidylserine probe PSS380 to label the entirety of the membranes (13, 17). We found that GFP-Ypt7 was enriched at the vertex ring of WT vacuoles as previously reported (Fig. 6, *A*–*C*). In contrast, GFP-Ypt7 accumulate poorly at *elo3*Δ vertices and was present at higher levels at the outer membrane edges relative vertices. This indicated that C26-SL were required for its localization to the vertex. This could be due to altered protein-protein interactions as seen with HOPS in pulldown experiments. To see if lower Ypt7 levels at *elo3*Δ vertices was indeed due to mislocalization and not to decreased expression, we compared the total levels of GFP-Ypt7 on WT and *elo3*Δ vacuoles by Western blotting. This showed that there was no difference in protein on isolated vacuoles (Fig. 6*D*). Vph1 was used as a loading control.

### The vertex enrichment of HOPS subunits is not altered on *elo3*Δ vacuoles

**Figure 7:**
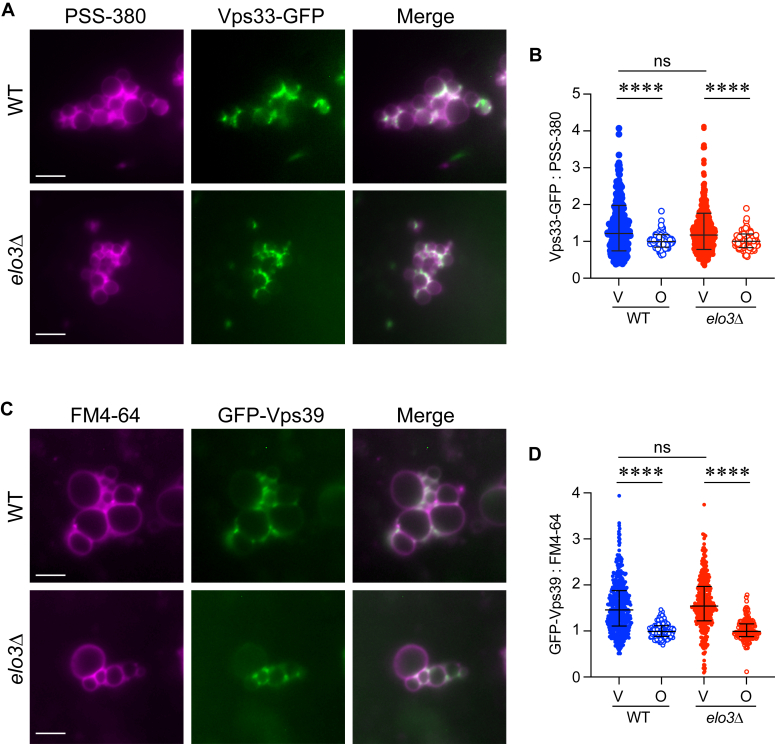
**HOPS localization to vertex domains is not affected on *elo3*Δ vacuoles.***A*, WT and *elo3*Δ vacuoles containing Vps33-GFP were analyzed for vertex enrichment as described above. Scale bar: 5 μm. *B*, quantitation of ratiometric of Vps33-GFP fluorescence intensities for V and O in panel C. Data was collected as described in (*B*). Error bars represent geometric means ± geometric SD. (n = 3). Significance was measured using one-way ANOVA for multiple comparisons [F (3, 1148) = 24.71, ∗∗∗*p* < 0.0001]. Tukey’s *post hoc* test for multiple comparisons was used for individual *p* values. ∗∗∗∗*p* < 0.0001; ns, not significant. *C*, WT and *elo3*Δ vacuoles containing GFP-Vps39 were analyzed for vertex enrichment as described above. Scale bar: 5 μm. *D*, quantitation of ratiometric of GFP-Vps39 fluorescence intensities for V and O in panel C. Data was collected as described in (*B*). Error bars represent geometric means ± geometric SD. (n = 3). Significance was measured using one-way ANOVA for multiple comparisons [F (3, 1110) = 92.40) = ∗∗∗*p* < 0.0001]. Tukey’s *post hoc* test for multiple comparisons was used for individual *p* values. ∗∗∗∗*p* < 0.0001; ns, not significant.

Due to the lack of Ypt7 at *elo3*Δ vertices, we asked if HOPS was also missorted to the microdomain. To test this, we used WT and *elo3*Δ vacuoles that harbored Vps33-GFP. Unlike the mislocalization of Ypt7 on *elo3*Δ vacuoles, Vps33-GFP enrichment at vertex sites was not affected (Fig. 7, *A* and *B*). Vps33 is part of the core Class-C complex that does not directly contact Ypt7. Thus, it was possible that HOPS subunits that do touch Ypt7 could be mislocalized. Both Vps41 and Vps39 directly contact Ypt7 on vacuoles (12, 88, 91). Recently, HOPS was found to be assembled from subcomplexes containing Vps11 and Vps39 (HOPS-2) with ones composed of Vps33, Vps16, Vps18 and Vps41 (HOPS-4) (92). To see if there was a separation of HOPS subunits on *elo3*Δ vacuoles we monitored GFP-Vps39. Similar to Vps33-GFP, we found that there was no difference in GFP-Vps39 enrichment on WT *versus elo3*Δ vacuoles (Fig. 7, *D* and *E*). The lack of an effect on HOPS localization on *elo3*Δ vacuoles while Ypt7 is missorted is consistent with the lack of interactions between HOPS and GST-Ypt7 in Fig. 5.

### Aureobasidin alters Ypt7 localization *in vivo* to mimic the *elo3*Δ phenotype

**Figure 8:**
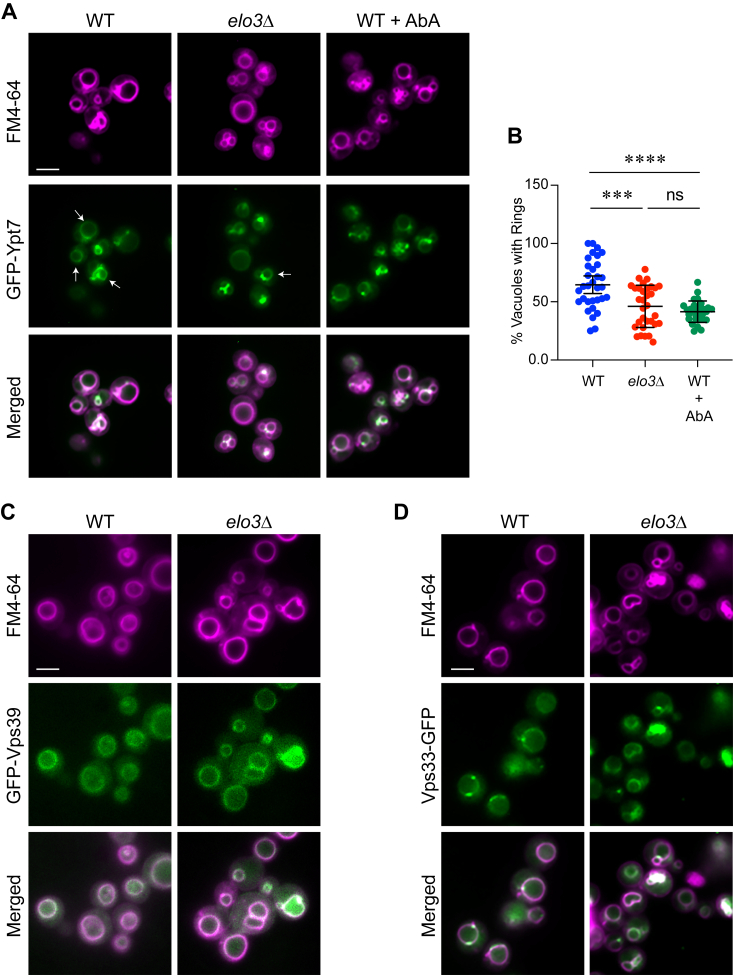
**Treating WT cells with Aureobasidin A mimics the altered GFP-Ypt7 localization seen in *elo3*Δ cells.***A*, wild type and *elo3*Δ yeast expressing GFP-Ypt7 were grown overnight in selective media and back diluted to ∼0.7 OD_600_ the next day. Cells were transferred to YPD broth and incubated for 4 h at 30 °C while shaking. WT cells were also treated with AbA. Cells were then incubated with 10 μM MDY-64 for 15 min in the dark before visualization. Images are representative of three separate trials. Arrows represent complete rings of GFP-Ypt7 around a vacuole. Scale bars: 4 μm. *B*, quantitation of the number of vacuoles with complete rings of GFP-Ypt7 per cell. Ten 63x fields were counted for a total of 200 to 600 vacuoles per condition/strain for each experiment. The number of vacuoles with complete GFP-Ypt7 in each field was converted to a percentage of total amount of vacuoles. Bars represent mean ± 95% CI. Significance was measured using one-way ANOVA for multiple comparisons [F (2, 90) = 16.00, ∗∗∗∗*p* < 0.0001]. Tukey’s *post hoc* multiple comparisons test was used for individual *p* values. ∗∗∗*p* < 0.0001, ∗∗∗∗*p* < 0.0001; ns, not significant. *C*-*D*, WT and *elo3*Δ yeast expressing GFP-Vps39 (*C*), and Vps33-GFP (*D*) were grown as described in panel A overnight and diluted to ∼0.7 OD_600_ the next day. Cells were transferred to YPD broth and incubated for 4 h at 30 °C while shaking. Cells were stained with FM4-64 and visualized by fluorescence microscopy. Images are representative of three separate trials. Scale bars: 4 μm.

We next asked if GFP-Ypt7 localization would be affected *in vivo* when sphingolipid production was inhibited. To do this we treated WT cells expressing GFP-Ypt7 with Aureobasidin A (AbA) and compared Ypt7 distribution in *elo3*Δ cells. In logarithmically growing cells GFP-Ypt7 was evenly distributed around the vacuole to form complete rings that overlap with FM4-64 (8, 17). We observed the same rings here, however, when cells were treated with AbA we found that GFP-Ypt7 formed fewer rings and with reduced intensity suggesting that SL production was needed for normal Ypt7 distribution (Fig. 8, *A* and *B*). The altered distribution of Ypt7 in WT treated cells with AbA was indistinguishable from the distribution of Ypt7 in *elo3*Δ further linking SL production and Ypt7. In addition to rings, Ypt7 in whole cells also collects in punctate domains that overlap with FM4-64 with some found within the vacuole lumen. It could be that mislocalized Ypt7 is located to puncta. Luminal puncta could likely due to post-fusion internalization of membrane fragments (89). Based on the role of SL in lipid raft formation and what we observed when we measured membrane fluidity, we propose that SL normally provide membrane stiffness needed for Ypt7 to localize where it functions during membrane fusion. In other words, vacuoles do not function properly when membranes are too fluid.

### Dibucaine treatment of WT vacuoles reproduces the *elo3*Δ phenotype

**Figure 9:**
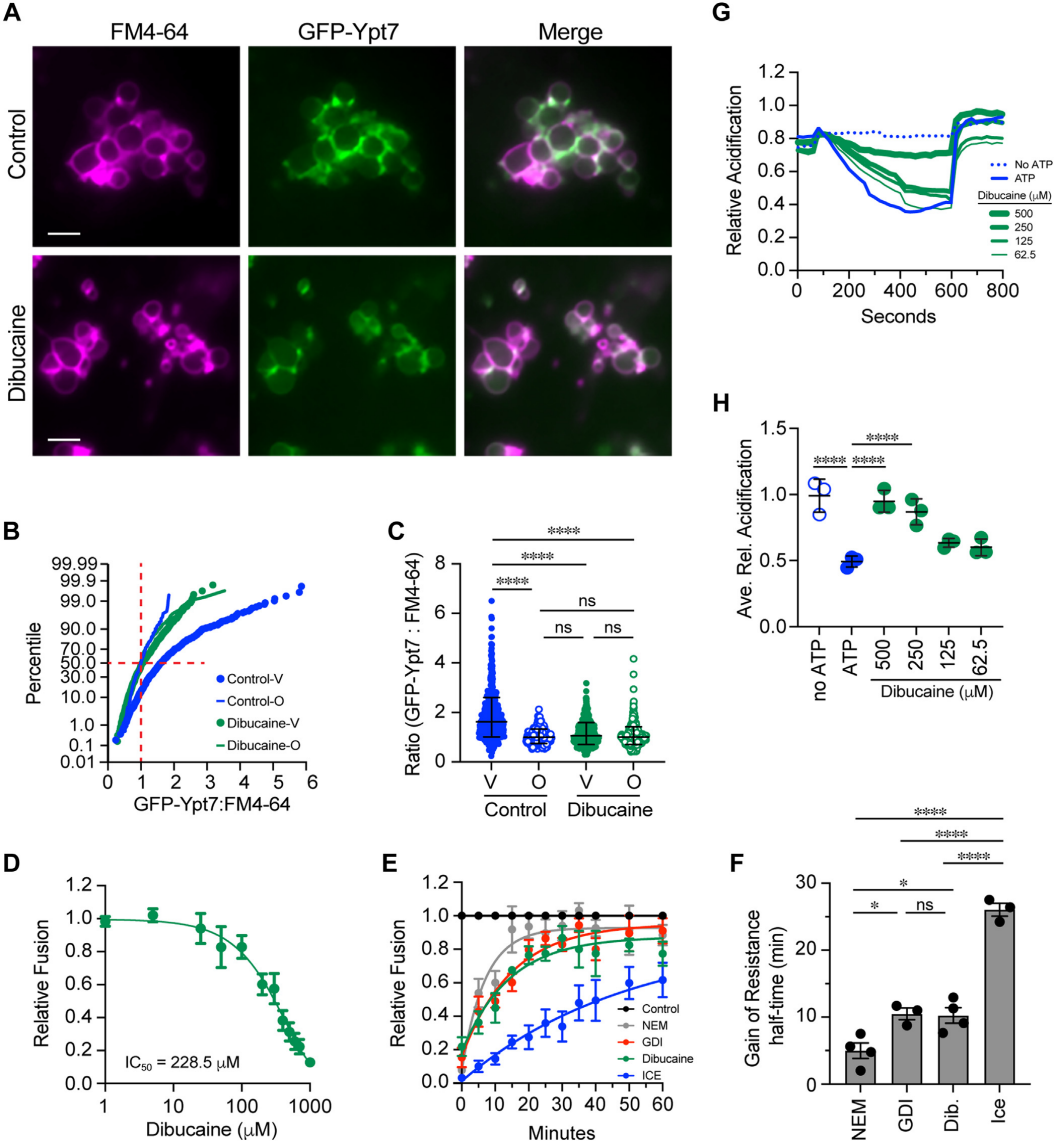
**Dibucainetreatment of WT reproduces the *elo3*Δ phenotype.***A*, docking reactions containing of WT vacuoles containing GFP-Ypt7 were performed in the with or without 250 μM Dibucaine. FM4-64 was added at the end of the reaction and docked vacuoles were visualized using fluorescence microscopy. Scale bar: 5 μm. *B*, cumulative distribution plot depicting the percentile values of GFP-Ypt7/FM4-64 ratio for vertex (V) and outer edge (O) and the effect of dibucaine. Each curve is comprised of pooled data points from three experiments. Each experiment used 10 to 15 clusters with ≥10 vacuoles per cluster (n > 400 vertices for each strain; n > 200 outer edge measurements for each strain) for each condition. *C*, geometric means ± geometric SD for the data in panel B. Significant differences were measured using one-way ANOVA for multiple comparisons [F (3, 1607) = 149.8, ∗∗∗∗*p* < 0.0001]. Tukey’s *post hoc* test was used for individual *p* values. ∗∗∗∗*p* < 0.0001; ns, not significant. *D*, fusion reactions were performed with WT vacuoles treated with a dosage curve of Dibucaine. IC_50_ was determined using Prism. Error bars represent mean ± SE. (n = 3). *E*, gain of resistance vacuole fusion assays were performed in the presence of buffer, 1 mM NEM, 2 μM GDI or 500 μM dibucaine. Fusion reactions were incubated for 120 min at 27 °C. Reactions were treated with reagents or placed on ice at the indicated time points. Fusion was normalized to the buffer-treated reactions at each time point. *F*, calculated half-times of inhibition from data in *E*. Significant differences were measured using one-way ANOVA for multiple comparisons [F (3, 10) = 63.4, ∗∗∗∗*p* < 0.0001]. Tukey’s *post hoc* test was used for individual *p* values. Error bars represent mean ± SE. (n = 4). ∗*p* < 0.05, ∗∗∗∗*p* < 0.0001, ns, not significant. *G*, vacuole acidification was determined for WT in the presence of dibucaine at the indicated concentrations. AO fluorescence was measured at 520 nm every 20 s. A reaction lacking ATP was used as a negative control. FCCP was added at 600 s to collapse the proton gradient. Fluorescence was normalized to initial intensities at the time of adding ATP and set to 1. *H*, quantitation of three experiments shown in *D*. Fluorescence intensities were averaged for each condition at 400 s. Significant differences were measured using one-way ANOVA for multiple comparisons [F (9, 20) = 19.31, ∗∗∗∗*p* < 0.0001]. Tukey’s *post hoc* test was used for individual *p* values. Error bars represent mean ± SE. (n = 3). ∗∗∗∗*p* < 0.0001.

Finally, we asked if artificially increasing membrane fluidity in WT vacuoles with dibucaine would reproduce the *elo3*Δ phenotype. We first tested the effects of dibucaine on the localization of GFP-Ypt7. WT vacuoles were treated with dibucaine and GFP-Ypt7 distribution was measured by fluorescence microscopy. We found that dibucaine inhibited GFP-Ypt7 localization to vertex rings in a manner that was indistinguishable from what occurred on *elo3*Δ vacuoles, demonstrating that dibucaine also inhibited optimal tethering and docking (Fig. 9, *A*–*C*). Panel B shows the cumulative distribution curves of combined experiments, depicting the mean ratio of GFP-Ypt7 fluorescence at vertices of untreated control vacuoles *versus* outer edges was greater that 1 and thus right-shifted. This also showed that the ratio of GFP-Ypt7 at the vertices of dibucaine-treated vacuoles was the same as the outer edge. Panel C shows geometric means and geometric standard deviations. Differences in GFP-Ypt7 fluorescence was not due to loss of protein on dibucaine treated vacuoles as shown by the Western blot in Fig. 6*C*.

Next, we tested the effect of dibucaine on fusion. We found that dibucaine inhibited vacuole fusion in a dose dependent manner, which further paralleled the *elo3*Δ phenotype (Fig. 9*D*). To verify if dibucaine inhibited at the tethering stage we performed a gain of resistance assay (31, 93). In this test inhibitors were added to individual reactions at different times starting at the beginning of the assay (T = 0 min) and at 10 min intervals for a total of 120 min (Fig. 9*E*). The first time point in each curve showed the maximum inhibition for each reagent. As fusion reactions passed through specific stages, they became resistant to stage-specific inhibitors. For instance, NEM was used to inhibit priming so reactions that have completed priming became resistant to the inhibitor. Similarly, GDI was used to inhibit vacuole tethering, which is demonstrated by the right-shift of the curve and increased half-time of resistance (Fig. 9*F*). Using this test, we found that reactions became resistant to dibucaine with a curve that overlapped with that of GDI, demonstrating that dibucaine inhibited during the tethering stage. This is consistent with the microscopy assay of GFP-Ypt7 distribution. Furthermore, the calculated half-time of resistance was similar to the one determined for GDI. Aside from docking and fusion, we found that V-ATPase activity was reduced on *elo3*Δ vacuoles. Thus, we tested if dibucaine could also affect vacuole acidification. Indeed, we found that vacuole acidification was reduced when treated with dibucaine (Fig. 9, *G* and *H*). Together these data showed that dibucaine could reproduce the defects we observed with *elo3*Δ, suggesting that a major function of C26-SL was to set the optimal fluidity for the vacuole fusion system to work.

We also tested whether dibucaine treatment of isolated vacuoles would reproduce the inhibited interactions between GST-Ypt7 and HOPS observed with *elo3*Δ vacuole extracts. However, when we performed the pulldown experiments with dibucaine-treated WT vacuoles we found that there was no effect on Ypt7-HOPS binding. This suggested that while the lack of C26-SL affects fluidity in a way that dibucaine can reproduce on isolated vacuoles, there exists at least one more defect that cannot be replicated after isolation. Further investigations will be needed to find additional C26-SL dependent factors that control vacuole fusion.

## Discussion

Studies have shown that C26 VLCFAs are found almost exclusively within sphingolipids at the amide bond position, leaving only trace amounts of free VLCFAs within the cell (45, 94). Therefore, we postulate that the observations outlined above stem from a lack of C26-SL, as opposed to a lack of free cerotic acid. This is bolstered by the observation that cells treated with sphingolipid biosynthesis inhibitors display aberrant vacuolar morphology that mimicked *elo3*Δ cells. WT yeast produce sphingolipids that most often (70–90%) contain a C26:0:1 (cerotic acid with no double bonds, and a single hydroxylation in the acyl chain), whereas yeast that lack Elo3 have been shown to produce non-canonical sphingolipids with shorter acyl chains (20–24 carbons). This is accompanied by a significant decrease in complex sphingolipid levels (51, 95). In contrast, the bulk of the glycerophospholipids, whose acyl chains are usually 16 or 18 carbons long, and typically contain one or two desaturated fatty acids (16:1 and/or 18:1) such as PI3P were not affected by the absence of C26-SL on the vacuole. Importantly, we found that yeast strains lacking C26 VLCFAs are unable to enrich the Rab Ypt7 at the vertex region and fail to support its interactions with HOPS, which is likely linked to increased membrane fluidity.

The observation that vacuoles isolated from cells lacking C26-SL are fusion deficient could arguably be attributed to one or both of the following: biophysical requirements of the membrane – such as interdigitation of acyl chains, or strong hydrogen bonding (96, 97); and/or sphingolipid-rich domains at vertex regions that serve as platforms for fusion protein concentration and localized activity (1, 58, 98). Apropos, others have found that VLCFAs can stimulate membrane fusion in plant cells during cytokinesis and with liposomes (73).

Lipids isolated from yeast can assemble into model membranes that separate into visibly distinct domains when they are labeled with fluorescent dyes with different lipid-partitioning properties (57). The domain that was found to be liquid-ordered-like (L_o_-like) was enriched in BODIPY-cholesterol, and this domain dissipated when the lipids were isolated from yeast lacking a functional Elo3. This lipid phase separation is also seen when yeast cells are grown to stationary phase. The vacuole displays regions that separate into distinct lipid domains *in vivo* (59, 99, 100). The domains are enriched in either the V-ATPase subunit Vph1 or Ivy1, a PI(3,5)P_2_-binding protein that interacts with Ypt7 and Vps33 (101, 102, 103), and they display properties that are indicative of differences in relative membrane fluidity. Vph1 colocalizes with a dye that partitions into liquid-disordered-like (L_d_-like) areas, while Ivy1 colocalizes with ergosterol in L_o_-like regions of the membrane. Vph1 localization was subsequently utilized as a negative marker in studies to show that the regulatory fusion lipids PI3P, PI4P, and PI(3,5)P_2_ tend to enrich in regions that lack Vph1 and intramembrane particles (104, 105), *i.e.* these known regulatory lipids enrich in the raft-like domains that also contain relatively high amounts of sphingolipids and sterols. It should also be noted that others have shown that the V-ATPase is activated upon the addition of exogenous, short-chain PI(3,5)P_2_ which stabilizes the V_O_-V_1_ complex, suggestive of a protein-lipid interaction between L_o_ and L_d_ residents (106). Similarly, we showed that overproduction of PI(3,5)P_2_ enhanced V-ATPase activity (33).

So how does this relate to Ypt7 localization to vertex sites? Membrane microdomains that contain cholesterol and sphingolipids can recruit proteins that are conjugated to saturated lipids such palmitic acid; however, these domains exclude proteins with unsaturated and/or branched lipid anchors including Rabs, which are covalently linked to two branched and unsaturated geranylgeranyl lipids (107, 108). This implies that proteins in raft-like domains can interact with non-raft proteins including Rabs. The notion of bridging L_o_ and L_d_ domains is supported by interactions of Ivy1, Vps33 and Ypt7 in yeast, as well as the interactions of Rab10 and flotillin in HeLa cells (102, 103, 109). Furthermore, disrupting microdomains with high doses of filipin to bind ergosterol causes Ypt7 to dissipate throughout membranes (13). The current study shows that the lack of C26 VLCFA redistributes Ypt7, but not the vertex enrichment of ergosterol, PI3P and HOPS. Together this suggests that the effects on Ypt7 are not necessarily due to raft *versus* non-raft recruitment, but instead linked to a related, but separate aspect of membrane dynamics such as fluidity. Changes in fluidity can affect protein-protein interactions. For example, extracting cholesterol with methyl beta-cyclodextrin inhibits interactions between flotillin and the alpha and beta subunits of a heterotrimeric G-protein in sphingomyelin-enriched detergent resistant Golgi complexes, while other interactions remained intact (110). This proposes that the raft environment itself is not required for interactions but may affect binding affinities. Thus, changes in fluidity might affect the retention of Ypt7 at vertices by altering interactions with binding partners such as HOPS and Ivy1.

While there is no evidence of membrane fluidity directly affecting the binding of Ypt7 and HOPS, other factors have been shown to affect their interaction. One possibility for disrupting this interaction could be linked to posttranslational modifications including HOPS phosphorylation by the casein kinase Yck3 (88, 91, 111, 112, 113). This would be consistent with other studies showing that casein kinases are activated by sphingolipids (114, 115). In rat synaptosomes, casein kinase two associates with detergent resistant microdomains and its activity is abolished when cholesterol is extracted (116). Thus, it is possible that Yck3 activity is altered on *elo3*Δ vacuoles leading to a disruption in the binding of HOPS and Ypt7. Zick and Wickner found that HOPS phosphorylation (P-HOPS) by Yck3 alters binding to Ypt7 (112). Specifically, they found that P-HOPS preferentially binds GTP-loaded Ypt7 *versus* GDP-Ypt7; however, the overall binding of P-HOPS to Ypt7 was sharply reduced when compared to Ypt7 binding by unphosphorylated HOPS. This indicates that Yck3 in general inhibits HOPS binding to Ypt7, which is in accord with reports by LaGrassa *et al.* showing that phospho-Vps41 does not bind GDP-Ypt7 (111). Together, this tells us that differences in Yck3 function in *elo3*Δ is not responsible for the mislocalization of Ypt7. This conclusion is supported by data showing that when *YCK3* is deleted, vacuole fusion becomes resistant to GDI (88, 111). We found that the GDI potently inhibited the fusion of WT and *elo3*Δ vacuoles with identical IC_50_ values, indicating that Yck3 is not playing a role in the altered fusion of *elo3*Δ vacuoles.

Could other posttranslational modifications be involved? While Vps41 is the target of Yck3, Vps39 has been found as a substrate in phosphoproteome studies that focused on the cyclin dependent kinase Cdk1 (117), and the DTT activated kinase Ire1 (118). Aside from phosphorylation, Vps39 is also methylated according to Wang *et al.* (119). However, the roles of these modifications remain unknown in HOPS function for now. Perhaps one of these modifications will shed light on the altered fusion of *elo3*Δ vacuoles.

Another possibility for the inhibited interaction between and HOPS and Ypt7 on *elo3*Δ vacuoles could be through altered protein-protein interactions. One prospect is connected to actin dynamics. Previous studies have shown the interplay between HOPS and Rab GTPases and actin remodeling. On yeast vacuoles, filamentous actin (F-actin) depolymerizes to globular actin (G-actin) early in the pathway and later accumulates at vertex sites and repolymerizes at a later stage of the pathway (120). Blocking F-to-G depolymerization with Jasplakinolide (JP) blocks HOPS from localizing to vertices while Ypt7 is unaffected (16). JP also blocks PI3P accumulation at vertices, which likely plays a role in the lack of HOPS (13). Furthermore, antibodies against Ypt7 or Vps33 blocks actin accumulation at vertices (15). Together, the connection between actin and vertex enrichment of Ypt7 and HOPS is clear. So, how does this connect to C26-SL? A study from Balguerie *et al.* found that when the Amphiphysin-like protein Rvs161 is deleted, actin fails to polymerize after salt shock, however, this was rescued when *ELO3 (SUR4)* was also deleted (121). Furthermore, Rvs161 is mislocalized in *elo3*Δ strains, and *elo3*Δ strains showed faster actin depolarization (ibid). This shows that while the total amount of actin is the same in WT and *elo3*Δ strains, the lack of VLCFAs or C26-SL causes actin polymerization related issues, which is essential for the completion of the fusion pathway through interaction with Rabs and HOPS. Therefore, it is not improbable that altered actin remodeling blocks the interaction between Ypt7 and HOPS. Moreover, it is likely that the altered actin in *elo3*Δ vacuoles cannot be replicated by treating isolated vacuoles with dibucaine and thus cannot reproduce the block in Ypt7-HOPS interactions shown in the WT pulldown. The link between actin and HOPS-Rab interactions is not limited to yeast vacuoles. In the primary cilium of polarized epithelia, studies have shown that Rab19 accumulate at the inner periphery of cortical actin clearing, and deleting Rab19 or the HOPS subunit Vps41 caused a defect in actin clearing and ciliogenesis (122).

## Experimental procedures

### Yeast strains and growth conditions

Yeast strains used in this study are found in Table 1. Yeast strains were grown in YPD (1% yeast extract, 2% peptone, 2% dextrose, or synthetic drop-out media (YNB, yeast nitrogen base lacking amino acids and ammonium sulfate, with amino acids, adenine sulfate, and uracil added at 76 mg/L, except leucine which was at 380 mg/L lacking the specified nutrient the specified nutrient. The *ELO3* coding sequence was deleted using homologous recombination by amplifying the *hphMX6* gene conferring hygromycin resistance from the vector pAG32 (Addgene) using the primers elo3F (5′-CGGCTTTTTTCCGTTTGTTTACGAAACATAAACAGTCATCTGTTTAGCTTGCCTTGTCC-3′) and elo3R (5′-TTTTTTCTTTTTCATTCGCTGTCAAAAATTCTCGCTTCCGA CACTGGATGGCGGCGTTA-3′). The PCR product designed for homologous recombination was transformed using lithium acetate as previously described (123), and cells were grown on YPD agar plates containing 200 mg/L hygromycin B. The construct was confirmed *via* sequencing. All plasmids were transformed into wild type and *elo3*Δ strains *via* electroporation and plated onto appropriate auxotrophic selection plates.

**Table 1:** Strains used in this study

Strain	Genotype	Source
BJ3505	*MATa pep4::HIS3 prb1-*Δ*1.6R his3-200 lys2-801 trp1*Δ*101 (gal3) ura3-52 gal2 can1*	(144)
DKY6281	*MATα leu2-3112 ura3-52 his3-*Δ*200 trp1-*Δ*901 lys2-801 suc2-*Δ*9 pho8*Δ*::TRP1*	(145)
SEY6210	*MATα leu2-3112 ura3-52 his3-*Δ*200 trp1-*Δ*901 lys2-801 suc2-*Δ*9*	(145)
GFP-Ypt7	*SEY6210, ypt7::HIS3 pRS304:GFP-Ypt7 (TRP1)*	(17)
RFY120	*DKY6281, elo3::hphMX6*	This study
RFY121	*BJ3505, elo3::hphMX6*	This study
RFY124	*SEY6210, ypt7::URA3 elo3::hphMX6 pRS304:GFP-Ypt7*	This study
Vps33-GFP	*DKY6281, VPS33-GFP*	(17)
RFY125	*DKY6281, elo3::hphMX6 VPS33-GFP*	This study
GFP-Vps39	*SEY6210, vps39::HIS3 pYlPlac211-GFP-VPS33 (TRP)*	(17)
RFY126	*SEY6210, vps39::HIS3 elo3::hphMX6 pYlPlac211-GFP-VPS33*	This study

### Live microscopy

General vacuolar morphology of live yeast was determined by growing cells overnight in YPD at 30 °C to mid-log phase. Cells were then diluted in fresh YPD to OD_600_ ∼ 0.4 and allowed to grow for 2 h with the addition of 5 μM Yeast Vacuole Membrane Marker FM4-64 (Thermo Fisher), before treatment with 0.0125 μg/ml aureobasidin A (Takara) or DMSO vehicle control for an additional ∼4 h of growth. Cells were then isolated by centrifugation (12000*g*, 1 min, RT) and resuspended in PBS buffer. After resuspension, 20 μl of cell suspension were then mixed 1:1 with 0.6% low-melt agarose warmed to 50 °C, and 10 μl of the mixture was placed on a glass slide and covered with a glass coverslip for viewing by fluorescence microscopy. For strains expressing a fluorescent construct, overnight growth was in SC dropout media for plasmid maintenance before cells were back-diluted in YPD on the day of visualization. Images were acquired using a Zeiss Axio Observer Z1 microscope equipped with an X-Cite 120XL light source, Plan Apochromat 63x oil objective (NA 1.4) and an AxioCam CCD camera. All images were analyzed with ImageJ software (NIH). FM4-64 images were visualized with a 42 HE CY3 shift-free filter set, Filipin was visualized with a 49 DAPI shift free filter set and GFP was visualized using a 38 HE EGFP shift-free filter set.

### Vacuole isolation and *in vitro* content mixing

Yeast vacuoles were isolated as described (124) with slight modifications. Cells were grown at 30 °C and shaking at 225 rpm in 1L YPD broth in a 2L Erlenmeyer flask to OD_600_ values of 0.7 to 1.1. Cells were then pelleted by centrifugation (3000*g*, 5 min, 24 °C) and washed with 50 ml of 100 mM TRIS-Cl, pH 9.4, 15 mM DTT, and incubated at 30 °C for 10 min. The cells were pelleted again as above and resuspended in 15 ml spheroplasting buffer (50 mM potassium phosphate buffer pH 7.5 and 600 mM sorbitol in YPD containing 0.2% dextrose) with 0.8 to 1.5 mg/ml oxalyticase per O.D. unit of cells. Recombinant oxalyticase was purified as described (125). Cells were incubated for 30 to 45 min at 30 °C and transferred to chilled oak ridge tubes before spinning for 10 min at 1000*g*, 4 °C. All other steps in vacuole isolation were identical as referenced. Content mixing reactions (30 μl) contained 3 μg each of vacuoles from BJ3505 (*pep4*Δ *PHO8*) and DKY6281 (*PEP4 pho8*Δ) backgrounds in fusion reaction buffer consisting of PIPES-sorbitol (PS) buffer (20 mM PIPES-KOH, pH 6.8, 200 mM sorbitol), 125 mM KCl, 5 mM MgCl_2_, ATP regenerating system (1 mM ATP, 0.1 mg/ml creatine kinase [Sigma], 29 mM creatine phosphate [Gold Biotech]), 10 μM CoA, and 280 nM recombinant Pbi2 (IB_2_, Protease B inhibitor) (126). Reactions were incubated at 27 °C and Pho8 activity was assayed in 250 mM Tris-Cl, pH 8.5, 0.4% Triton X-100, 10 mM MgCl_2_, 1 mM *p*-nitrophenyl phosphate (MP Biomedicals). Fusion was monitored as the absorbance at 400 nm from *p-*nitrophenolate production through phosphatase activity. Vacuole fusion was inhibited at the priming stage with N-ethylmaleimide (NEM) (9, 93). As a control for Ypt7 dependent tethering we used recombinant GDI (GDP dissociation inhibitor) to extract GDP-bound Ypt7 to block further tethering. Recombinant GDI with a C-terminal chitin binding peptide was produced as described and dialyzed against 125 mM KCl in PS buffer (127).

### Lipid mixing

Lipid mixing assays to track outer leaflet fusion (hemifusion) were performed as previously described (128, 129). Vacuoles were isolated from BJ3505 WT and BJ3505 *elo3*Δ yeast, and 300 μg of vacuoles (by protein) were labeled by incubating with 400 μl of a 150 μM rhodamine B DHPE (1,2-dihexadecanoyl-sn-glycero-3-phosphoethanolamine) (RhPE/Lissamine rhodamine; ThermoFisher) in PS buffer while nutating for 10 min at 4 °C. Following labeling, 800 μl of 15% Ficoll PM 400 (Cytiva) (diluted in PS buffer) was mixed with the vacuole-RhPE suspension and transferred to 11 × 60 mm polyallomer ultracentrifuge tube, overlaid with 1.2 ml of 8% Ficoll, 1.2 ml of 4% Ficoll, and 0.5 ml of PS buffer (0% Ficoll). Vacuoles were separated from free dye by centrifugation (100,000*g*, 25 min, 4 °C, SW-60 Ti rotor, Beckman L8-80M Ultracentrifuge) and recovered from the 0%–4% interface. Lipid mixing reactions (90 μl) contained 2 μg of labeled vacuoles and 16 μg of unlabeled vacuoles in reaction buffer. Reaction mixtures were transferred to a black, half-volume, 96-well flat-bottom microtiter plate (Greiner Bio-One), and fluorescence from dequenching was monitored (λ_ex_ = 544 nm, λ_em_ = 590 nm) in a POLARstar Omega BMG Labtech plate reading fluorometer. Readings were taken every minute for 60 min at 27 °C. At the end of incubation each well was treated with 1% TX-100 to solubilize membranes to reach the maximum fluorescence per well. Dequenching due to fusion was calculated as a percentage of the maximum fluorescence at each time point. The data was then normalized to the starting fluorescence set to 1.

### Vacuole acidification

V-ATPase acidification of vacuoles was performed as previously described (33, 83, 130). *In vitro* acidification reactions (60 μl) contained 18 μg BJ3505 background vacuoles, reaction buffer, ATP regenerating system, 10 μM CoA, 283 nM Pbi2, and 15 μM acridine orange (Sigma). Reactions were pipetted into a nonbinding black half-volume 96-well flat-bottom plate. ATP regenerating system or PS buffer was added, and reactions were incubated at 27 °C. Acridine orange fluorescence was measured in a fluorescence plate reader with the excitation at 485 nm and emission at 520 nm. Reactions were initiated with the addition of ATP regenerating system following the initial measurement. After decreases in fluorescence plateaued (400–600 s), we added 30 μM FCCP (Carbonyl cyanide-4-(trifluoromethoxy) phenylhydrazone) (Cayman Biochemical) to collapse the proton gradient and restore acridine orange fluorescence. Each trace was normalized to the fluorescence intensities at the time of adding ATP and set to 1.

### Western blotting

Upon isolation of vacuoles, 2 μg of total protein from each vacuole type was solubilized in 5x Laemmli buffer, resolved by SDS-PAGE, and transferred to nitrocellulose for immunoblotting. Rabbit antibodies against Vam3, Vam7, Vti1, Ykt6, Sec17, Sec18, Vps18, Vps41, Vps39, Vps16, Vps33, Ypt7, Vac8, Pho8 and Actin were prepared as previously described (10, 11, 87, 131, 132, 133, 134, 135, 136, 137, 138, 139). Mouse anti-Vph1 was from Abcam (ab113683), and Mouse anti-CPY (Carboxypeptidase Y) was from Thermo-Fisher (A-6428). Goat anti-rabbit IgG (H  + L) secondary antibody DyLight 650 (84,546) conjugate or Goat anti-mouse IgG (H  + L) secondary antibody DyLight 650 (84,545) conjugate were from Thermo-Fisher. Fluorescence was measured with an Azure 400.

### Tethering and docking

Isolated vacuoles were subjected to docking assays as previously described (13, 17) with slight modifications. Tethering reactions (30 μl) contained 6 μg of vacuoles isolated from the indicated strains in fusion reaction buffer modified for docking conditions (PS buffer, 100 mM KCl, 0.5 mM MgCl_2_, 0.33 mM ATP, 13 mM creatine phosphate, 33 μg/ml creatine kinase, 10 μM coenzyme A, and 280 nM IB_2_). Tethering was monitored by counting the number of vesicles per cluster using WT or *elo3*Δ vacuoles. As a control for Ypt7 dependent tethering we used recombinant GDI. A percentage of isolated vacuoles become tethered during the preparation so complete cluster disruption is not possible.

### Vertex microdomain formation

Measuring the vertex enrichment of factors during tethering and docking was performed with vacuoles from cells expressing GFP fusion proteins or labeled with lipid binding probes. To track GFP-Ypt7 localization reactions were incubated under docking conditions as described above and stained with 4 μM PSS-380 or FM4-64 prior to examination (13, 16). PSS-380 labels the bulk lipid phosphatidylserine (a gift from Drs. Smith and Koulov, Notre Dame University, South Bend IN) (140). Ergosterol vertex enrichment was monitored with the fluorescent polyene macrolide Filipin III (5 μM) (Cayman) and stained with FM4-64. To localize the distribution of PI3P on vacuoles, reactions were incubated with the PI3P binding protein domain FYVE conjugated to the fluor Cy3 (0.3 μM) and the non-specific membrane dye MDY-64 (0.5 μM). Recombinant GST-FYVE was produced as described and dialyzed against 125 mM KCl in PS buffer (85), and conjugated to mono-reactive NHS ester Cy3 dye following manufacturer’s instructions (Cytiva). Docking reactions were incubated with Filipin or Cy3-FYVE as previously described (8, 13). Following incubation at 27 °C for 20 min, reactions were mixed with 20 μl of 0.6% low melt agarose in PS buffer melted at 50 °C and cooled to prior to mixing with vacuoles. Next, 20 μl aliquots were mounted on pre-chilled slides and observed by fluorescence microscopy. Exposure times were set using WT vacuoles for each fluorescence channel and scripts acquired non-specific images followed by specific reporters. This ensures that bleaching is consistent to negate it as a factor in calculating intensity ratios. Exposure times were held constant within an experiment.

Images were analyzed using ImageJ software (NIH). Vertex enrichment was determined by first measuring maximum fluorescence intensity in each channel at each contact point between membranes, *i.e.*, vertex domain within a cluster. Next, fluorescence intensity was measured in each channel at outer membrane domains where vacuoles are not in contact with other membranes. The ratio of specific (*e.g.,* GFP) to non-specific (*e.g.,* FM4-64) was determined for vertices and outer membrane domains and compared for relative enrichment. Measurements for each condition were taken of 15 to 20 clusters to yield 100 to 300 vertices for each condition/strain per experiment. Data from multiple experiments are combined in column plots showing individual values as well as the geometric means and geometric standard deviation for each condition. One-way ANOVAs and Tukey’s *post hoc* analysis for multiple comparisons were used to measure significance. *p*-values <0.05 were considered significant.

### GST-Ypt7-HOPS interactions

GST-Ypt7 was purified and loaded onto glutathione agarose for co-precipitation analyses as previously described with minor modifications (88, 90). *E. coli* BL21(DE3) cells were grown in auto-induction media (11.33 g Na_2_HPO_4_-7H_2_O dibasic, 3 g KH_2_PO_4_, 20 g tryptone, 5 g yeast extract, 5 g NaCl in per liter) for 18 h, at 37 °C, with shaking at 220 rpm (141). Cells were harvested by centrifugation, washed in cold wash buffer (50 mM Tris-Cl, pH 8, 150 mM NaCl, 0.01% 2-mercaptoethanol, 5 mM MgCl_2_, 1 mM PMSF, and 5 μM leupeptin hemisulfate, lysed by sonication, and the lysate was separated from debris by ultracentrifugation (Type 70 Ti fixed angle rotor, 200,000*g*, 30 min, 4 °C). The clarified lysate was loaded onto glutathione agarose resin, washed with wash buffer ten column volumes of wash buffer, and GST-Ypt7 was eluted with one resin-bed volume of elution buffer composed of wash buffer containing 10 mM reduced glutathione.

Following purification, 25 μg of GST-Ypt7 was loaded onto 250 μl glutathione resin equilibrated with solubilization buffer (SB: 25 mM HEPES-KOH, pH 7.4, 150 mM NaCl, 10% glycerol, 0.5% Triton X-100, 5 mM MgCl_2_, 0.01% 2-mercaptoethanol, 1 mM PMSF) and charged with 5 mM GTP as previously described (90). The resin was nutated at 4 °C for 30 min then washed 3X with SB. Fusion reactions (6X) containing vacuoles isolated from WT or *elo3*Δ were incubated for 60 min at 27 °C. Next, vacuoles were reisolated by centrifugation (16,000*g*, 10 min, 4 °C) and resuspended in 200 μl SB and incubated on ice for 10 min. Insoluble debris was pelleted by centrifugation and 180 μl of the supernatant was transferred to a new chilled tube. Ten percent (18 μl) of the extract was removed and transferred to a new tube as the input, mixed with SDS-PAGE loading buffer and boiled for 5 min. The remaining 162 μl of lysate was mixed with 20 μl of GST-Ypt7 loaded glutathione beads and nutated for 2 h at 4 °C. After binding the beads were sedimented (2040*g*, 1 min, 4 °C) and the supernatant was aspirated after which the beads were washed 3X with SB. Proteins were eluted by boiling with SDS-PAGE for 5 min. The beads were then pelleted by centrifugation and supernatants were transferred to new tubes as the pulldown. Samples were submitted to SDS-PAGE and immunoblotting for GST-Ypt7 and the HOPS subunits Vps33 and Vps18.

### Membrane fluidity assay

Merocyanine 540 (Sigma) was used as previously described (78, 142, 143). Briefly, vacuoles isolated from BJ3505 WT or *elo3*Δ cells (18 μg total protein diluted up to 54 μl in PS buffer for a 0.5 mg/ml final concentration) were mixed with 6 μl of 100 μM MC540 in PS buffer (Stock MC540 was at 125 mM in DMSO) for a total volume of 60 μl and a final concentration of 0.2% DMSO. These mixtures were incubated in the dark for 20 min before re-isolating the vacuoles by centrifugation (2348*g* for 5 min at room temp) and resuspended in 60 μl PS buffer containing 2 mM ATP, 2 mM MgCl2, 125 mM KCl and 10 μM CoA. Resuspended mixtures were put in a black half-volume, flat-bottom 96-well plate and endpoint fluorescent emission was measured (λ_ex_ = 530 nm, λ_em_ = 585 nm, CLARIOStar BMG Labtech). Separate samples were treated with 1% Triton X-100 to solubilize membranes to release MC540 to determine maximum fluorescence at 585 nm.

### Statistical analysis

Results were expressed as the mean ± SEM, mean ± 95% confidence interval (CI), or geometric mean ± SD as needed. Experimental replicates (n) are defined as the number of separate experiments. Statistical analysis was performed by unpaired two-tailed *t* test or One-Way ANOVA for multiple comparisons using Prism 10 (GraphPad, San Diego, CA). Statistical significance is represented as follows: ∗*p* < 0.05, ∗∗*p* < 0.01, ∗∗∗*p* < 0.001, ∗∗∗∗*p* < 0.0001. Tukey, Dunnett or Šidák’s *post hoc* analysis were used for multiple comparisons and individual *p*-values.

## Data availability

All data generated or analyzed during this study are available upon request. Addition data sharing information is not applicable to this study.

## Conflict of interest

The authors declare that they have no conflict of interest.
